# Scaling law for electrocaloric temperature change in antiferroelectrics

**DOI:** 10.1038/srep19590

**Published:** 2016-01-22

**Authors:** S. Lisenkov, B. K. Mani, E. Glazkova, C. W. Miller, I. Ponomareva

**Affiliations:** 1 Department of Physics, University of South Florida, Tampa, Florida 33620, USA; 2 School of Chemistry and Materials Science, Rochester Institute of Technology, Rochester, NY 14623, USA

## Abstract

A combination of theoretical and first-principles computational methods, along with experimental evidence from the literature, were used to predict the existence of a scaling law for the electrocaloric temperature change in antiferroelectric materials. We show that the temperature change scales quadratically with electric field, allowing a simple transformation to collapse the set of Δ*T*(*E*) onto a single curve. This offers a unique method that can be used to predict electrocaloric behavior beyond the limits of present measurement ranges or in regions where data are not yet available.

The electrocaloric (ECE) effect has received much attention in recent years following the discovery of giant ECE in some ferroelectrics^1^,^2^,^3^,^4^,^5^,^6^,^7^,^8^. This effect is associated with a reversible change in temperature under adiabatic application of an electric field. Alternatively, the effect can be described by a reversible entropy change under isothermal application of the electric field. The potential of ferroelectrics to exhibit giant ECE stems from the large values of both dielectric susceptibility and pyroelectric constants in these materials. Antiferroelectrics (the antipolar counterparts of ferroelectrics) do not exhibit any spontaneous polarization, but could transition into a ferroelectric phase under the application of an electric field^9^. Combined with their large dielectric constants (e.g., more than 3000 in PbZrO_3_), the absence of a spontaneous polarization and associated vanishing pyroelectric constants are likely to result in an ECE that is dramatically different from the one in ferroelectrics. One example is the existence of the inverse (negative) ECE in PbZrO_3_^10^,^11^. The inverse ECE is associated with an adiabatic cooling (rather than heating) upon the application of an electric field. The purpose of this work is to predict the existence of a scaling law for the low-field electrocaloric temperature change in antiferroelectrics. Such a tool will help guide future materials explorations and evaluations because it can allow the magnitude of the electrocaloric effect to be predicted using experimentally accessible measurement protocols. This is an important tool, particularly when the fabrication of novel materials may not be fully optimized.

The subject of scaling laws for the magnetic entropy change in magnetocaloric materials has received much attention in recent years^12^,^13^. It is well established that magnetic materials that undergo second order magnetic phase transitions exhibit universal behavior for Δ*S*_*M*_(*T*). This in turn leads to scaling, which is usually understood as the collapse of the set of Δ*S*_*M*_(*T*) curves associated with different magnetic field changes. Here *S*_*M*_ and *T* are the magnetic entropy and temperature, respectively. The underlying cause of such behavior is the universality associated with critical phenomena that also serves as a foundation for theoretical derivations^14^. The utility of the scaling behavior in magnetism includes being able to extrapolate the magnetocaloric response at temperatures and fields not available in the laboratory, reduction of the experimental noise, correcting the influence of non-saturating conditions, detection and elimination of contributions from minority magnetic phases^13^.

Unlike magnetic materials, ferroelectrics and antiferroelectrics usually exhibit first order or first-order-like phase transitions. Naively, this seems to rule out the possibility to observe universal behavior or scaling laws. Indeed, we are not aware of any reports of scaling behavior of electrocaloric entropy or temperature change. The purpose of this article is to use a combination of macroscopic themodynamics and first-principles-based direct computations in order to: i) study the intrinsic electrocaloric effect in antiferroelectric PbZrO_3_ for a wide range of temperatures and electric fields; ii) predict the existence of a scaling law for electrocaloric Δ*T* in this material; iii) establish the origin of such an unexpected finding; and iv) demonstrate the relevance of our findings to the experimental measurements reported in the literature.

We begin by considering ECE in antiferroelectrics from the thermodynamics point of view. The Maxwell relation predicts that 
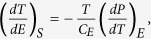
 where *P*, *E* and *C*_*E*_ are the macroscopic polarization, electric field, and volumetric heat capacity, respectively. Under low electric fields, the polarization in antiferroelectrics (and nonpolar phases of ferroelectrics) can be written as *P* = *εε*_0_*E*, where *ε* is the dielectric constant. This allows us to rewrite the Maxwell relation as 
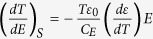
. This predicts that the ECE in linear dielectrics is proportional to the temperature derivative of the dielectric constant. For a typical antiferroelectric, the temperature derivative of the dielectric constant changes sign at the transition temperature^9^, which explains the experimentally observed sign change of the ECE in PbZrO_3_^10^,^11^. The typical electrocaloric change in temperature (Δ*T*) is rather small compared to *T*, which allows us to further approximate the Maxwell relation as 
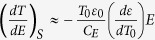
, where *T*_0_ is the temperature before the application of the electric field. Integrating the differential equation under the assumption of constant heat capacity^1^,^11^ yields the ECE temperature change 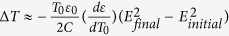, which is quadratic in electric field. Note that the same expression can be derived from the phenomenological Landau-Devonshire theory^15^. This equation suggests the possibility to collapse Δ*T*(*E*) onto a single curve. Indeed, one can choose a control curve 
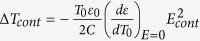
, where we set the initial electric field to zero. All other Δ*T*(*E*) curves can now be collapsed onto the control curve by using the transformation 
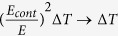
. It is worth emphasizing that the above relations and the associated analysis are only valid for Δ*T*/*T* ≪ 1 and electric fields that do not significantly affect *C*. As long as these requirements are met the collapse of Δ*T*(*E*) curves should occur for any linear dielectric. Note, that in ferroelectric phase the polarization is *P* = *P*_*spont*_ + *εε*_0_*E*, where *P*_*spont*_ is the spontaneous polarization. Therefore, the transformation 
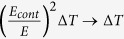
 can no longer be used to collapse Δ*T*(*T*) on the same curve.

Next we test the findings from the macroscopic thermodynamics against the predictions from first-principles direct ECE simulations. More precisely, we aim to establish whether the ECE temperature change is indeed quadratic in the electric field, and whether the points from different Δ*T*(*E*) dependencies can be collapsed onto a single curve. Note that our atomistic ECE simulations utilize the direct method to obtain caloric temperature change^16^,^17^, which does not require the use of Maxwell relations or any other elements of macroscopic thermodynamics. Therefore, these simulations are well suited to test the predictions from the thermodynamic theory.

We simulate bulk PbZrO_3_ that is modeled by a 16 × 16 × 16 supercell periodic along all three Cartesian directions. The total energy of the supercell is given by the first-principles effective Hamiltonian of Ref. 18. The degrees of freedom for the Hamiltonian include local modes that are proportional to the dipole moment in the unit cell, oxygen octahedron tilts about pseudocubic axes that describe oxygen octahedron rotation, and strain variables tensors that are responsible for the mechanical deformations of a unit cell. Note that the unit cell here refers to a five atom cell of cubic perovskite. The energy of the PbZrO_3_ Hamiltonian includes dipole-dipole interactions, short-range interactions, on-site self energies, elastic energy, coupling energy between the degrees of freedom and the interaction between the local modes and electric field. The Hamiltonian correctly reproduces many of the electrical and thermodynamical properties of PbZrO_3_^18^. In particular, it accurately predicts the antiferroelectric phase transition and the dipole pattern associated with it, electric hysteresis loops, and PbZrO_3_ behavior under pressure. Prior to ECE computations, the simulated sample was annealed from 1200 K to 5 K in steps of 5 K using the canonical Monte Carlo simulations. Thus equilibrated supercells were used in direct simulations of the ECE in the framework of the adiabatic Monte Carlo approach proposed in refs 16,17 Technically, the electric field directed along either [100] or [110] or [111] direction was first applied and then removed slowly at a rate of 150 V/m per one Monte Carlo sweep to ensure reversibility. No significant difference in Δ*T* was found for the different orientations of the electric field. The electrocaloric temperature was computed as a function of the applied field.

To estimate the electric fields associated with an antiferroelectric (AFE) to ferroelectric (FE) phase transition we compute the field-cooled *P*(*T*) dependencies given in Fig. 1(c). The curves indicate that the AFE-FE phase transition occurs under an electric field value of 1750 kV/cm. The curves are in qualitative agreement with the experimental data for La-doped Pb(ZrTi)O_3_ thin films^11^. Quantitatively, the Curie point and the electric field associated with the AFE-FE phase transition are overestimated in computations. This is in part due to the fact that we simulate an undoped and defect-free sample. Doping and defects are known to substantially lower the coercive fields in PbZrO_3_^19^,^20^, and to impact phase transitions in magnetic systems^21^.

We first focus on the ECE in the AFE phase and, therefore, apply electric fields of up to 1500 kV/cm. Figure 1(a,b) reports the electrocaloric Δ*T* as a function of the applied electric field. In agreement with experimental measurements^10^,^11^, we find a negative Δ*T* below the Curie point and a positive Δ*T* above the Curie point (946 K in computations). Remarkably, the electrocaloric Δ*T* obtained from the direct computations demonstrate a quadratic dependence on the electric field, in agreement with the predictions from thermodynamics and the experimental measurements of ref. 11. We have verified that the electrocaloric Δ*T* is quadratic in the electric field in the entire range of temperatures and electric fields associated with the AFE phase of PbZrO_3_. It should be noted that macroscopic thermodynamics predicts the quadratic dependence of Δ*T* on *E* for any dielectric in the low field limit. This is in agreement with the recent experimental measurements on relaxor ferroelectric ceramics^22^.

Figure 1(d) shows the computational Δ*T*(*T*) dependencies for a few select fields along with a prediction from the quadratic fit to the Δ*T*(*E*) data. We notice that the ECE remains negative for all temperatures below the Curie point and increases in magnitude as the transition temperature approaches. Above the Curie point the ECE is positive. The data are in qualitative agreement with the experimental results of ref. 11. Note that in experiments Δ*T*(*T*) is continuous at the Curie temperature, while computationally there exist some discontinuities. These discontinuities are due to the thermal and electric hysteresis associated with the first-order phase transition in bulk PbZrO_3_.

Next we turn to the possibility of collapsing the electrocaloric Δ*T*(*T*) dependencies onto a single curve. We choose data for the field 1400 kV/cm for the control curve because the PbZrO_3_ remains AFE below this field and exhibits electrocaloric Δ*T* that is quadratic in the electric field. The data for other fields (given in Fig. 1(d)) are then rescaled according to the transformation 
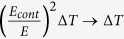
. Figure 2(a) gives the resultant curves that all collapse onto the control set. Our direct simulations confirm the existence of the scaling law for the low-field electrocaloric Δ*T*(*T*) in antiferroelectrics predicted from the macroscopic thermodynamics. It should be noted that our computations suggest that in defect-free PbZrO_3_ the scaling behavior occurs for all the fields below the critical field associated with the AFE-FE phase transition.

One of the advantages of the scaling law is the possibility of predicting the electrocaloric Δ*T* for the electric fields and temperatures for which the data are not available or for ranges of fields and temperatures that may be difficult to obtain in experiments, particularly when materials fabrication has not been optimized. Figure 2(b) gives the electrocaloric Δ*T* in PbZrO_3_ from the direct experimental measurements of ref. 10. We use the experimental set with the largest number of data points (the 60 kV/cm data set) as the control set for the scaling model. The scaling transformation 
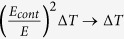
 is then applied to predict Δ*T*(*T*) for the fields of 20, 40, 80, 100 and 150 kV/cm. The predictions are shown by the lines in Fig. 2(b). Solid lines give the scaling model predictions in the regions where the experimental data are available, while dashed lines correspond to the electric fields and/or temperatures where the direct measurements do not exist. Note, that the curves are cut off where the fields may be high enough to induce a transition into a FE phase. We first notice a remarkable agreement between the predictions from the scaling model and the actual measurements for fields below the control one. For larger fields, some deviations occur which is to be expected since the model is most accurate for the electric fields low enough to maintain the AFE phase of the material and constant heat capacity. Secondly, we notice that the scaling model allows us to substantially extend the reach of the experimental measurements, as indicated by the dashed lines in Fig. 2(b). The predictions from the extended region could be qualitatively compared with experimental data for La-doped Pb(ZrTi)O_3_ thin films^11^ (see open symbols in Fig. 2(b)), which were obtained using the indirect approach. This comparison underscores the reliability of the scaling model. We conclude that the predictions from the macroscopic thermodynamics and direct first-principles-based computations, together with the experimental measurements from the literature, offer strong evidence for the existence of the scaling law for the low-field electrocaloric Δ*T*(*T*) in antiferroelectrics.

The existence of the scaling law in a material with a first-order phase transition is a rather striking result. Indeed, it was demonstrated in magnetism that magnetic materials that undergo first-order phase transition do not follow the universal scaling in their polar phases^12^. At the same time, this seeming controversy can be resolved by realizing that the scaling model proposed in this work has an entirely different origin. Indeed, the scaling law presented here originates from the linearity of the polarization response to the electric field, with the field being low enough to satisfy the conditions Δ*T*/*T* ≪ 1 and *C* ≈ constant, as well as the absence of spontaneous polarization. This scenario can be realized in nonpolar phases of electric and magnetic materials. Indeed, universal scaling was observed in nonpolar phases (above the Curie point) of magnetic materials with both second and first-order phase transitions^12^.

The existence of the scaling law is predicted in the AFE phase of antiferroelectrics. It is instructive to verify that the scaling law breaks down upon a field induced transition into a FE phase. To address this, we simulate the application of electric fields that are larger than the critical field associated with the AFE-FE phase transition. Figure 3 shows the *P*(*E*) and Δ*T*(*E*) dependencies from one such simulation. The initial application of the electric field in the AFE phase of PbZrO_3_ (region 1–2 in the figure) results in a temperature decrease (negative ECE). Further increasing the electric field (region 2–3) induces a first-order AFE-FE phase transition and results in an irreversible temperature increase. In the ferroelectric phase (region 3–4–5) the material’s response to the electric field changes dramatically. The electrocaloric Δ*T* now increases with the electric field (positive ECE). Positive ECE in the FE phase of antiferroelectric thin films was previously reported in ref. 1. Interestingly, however, in the FE regime the electrocaloric Δ*T* is linear in the electric field, unlike the AFE regime where Δ*T* is quadratic in the field. Upon the FE-AFE transition (region 5–6) the material experiences a second irreversible temperature increase due to the structural phase transition. Finally, PbZrO_3_ reenters the regime associated with the negative ECE on returning into the AFE phase (region 6–7). Our computational data demonstrate that the field induced AFE-FE phase transition is associated with irreversible heating, a change in the sign of ECE, and departure from the quadratic dependence of the electrocaloric Δ*T* on the electric field. The latter point signals that the scaling law will no longer be obeyed. Interestingly, this finding seems to suggest another possible application of the scaling law model. In such an application a failure of an AFE material to follow the scaling law indicates the presence of minority FE phases.

In summary, on the basis of macroscopic thermodynamics we proposed the scaling law for low-field electrocaloric Δ*T*(*T*) in antiferroelectrics. The existence of such a law was computationally demonstrated in antiferroelectric PbZrO_3_ from first-principles-based simulations. Application of the scaling model to experimental data from the literature provided further evidence for the existence of the scaling law for low-field electrocaloric Δ*T*(*T*). Potential applications of the scaling law include extrapolation to temperatures and fields for which data are not available, screening of novel materials performance, and the detection of minority phases^13^.

## Additional Information

**How to cite this article**: Lisenkov, S. *et al*. Scaling law for electrocaloric temperature change in antiferroelectrics. *Sci. Rep.* **6**, 19590; doi: 10.1038/srep19590 (2016).

## Figures and Tables

**Figure 1:**
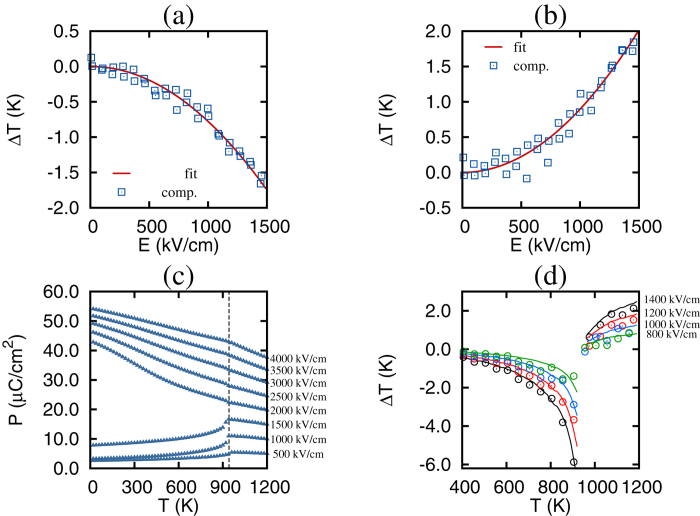
Dependence of the electrocaloric change in temperature on the electric field for T = 690 K (**a**) and T = 1050 K (**b**). Symbols represent computational data, while the solid line gives the fitting curve Δ*T* = *αE*^2^. (**c**) The dependence of polarization on temperature from annealing simulations under an applied electric field. (**d**) Temperature dependence of electrocaloric Δ*T* for select values of electric field. Symbols give computational data, while solid lines represent the predictions from the quadratic fit to Δ*T*(*E*) data.

**Figure 2:**
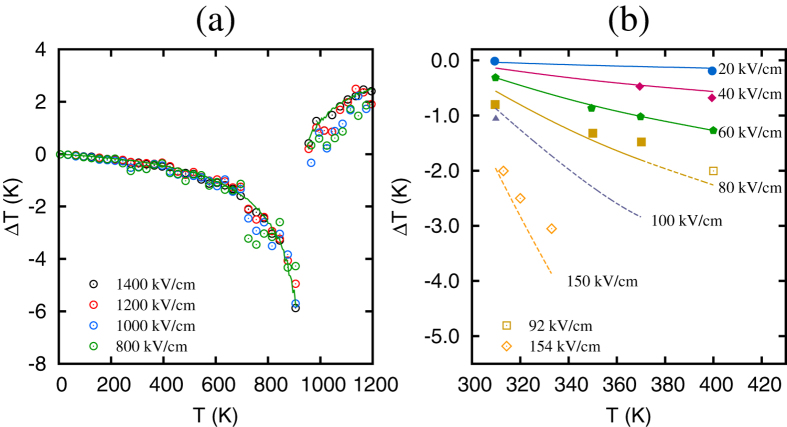
(**a**) Scaled curves obtained by applying the transformation 
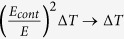
 with *E*_*cont*_ = 1400 kV/cm. Symbols give computational data, while solid lines represent the predictions from the quadratic fit to Δ*T*(*E*) data. (**b**) Electrocaloric change in temperature as a function of the temperature in PbZrO_3_ taken from the experimental data of ref. 10 (filled symbols). Lines give the predictions from the scaling model when using *E*_*cont*_ = 60 kV/cm. Dashed lines indicate the regions where the experimental data are not available. Open symbols give the experimental data for La-doped Pb(ZrTi)O_3_ thin films^11^

**Figure 3:**
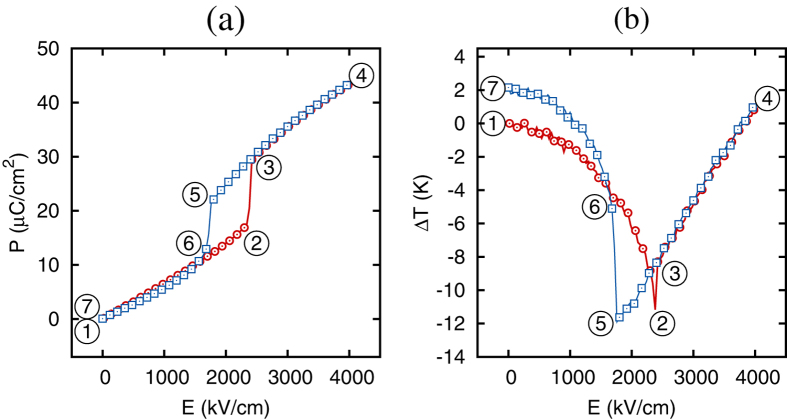
Dependence of the polarization (**a**) and electrocaloric temperature change (**b**) on the electric field applied along [110] direction at *T* = 800 K. Circles (squares) give the data upon the application (removal) of the electric field.
